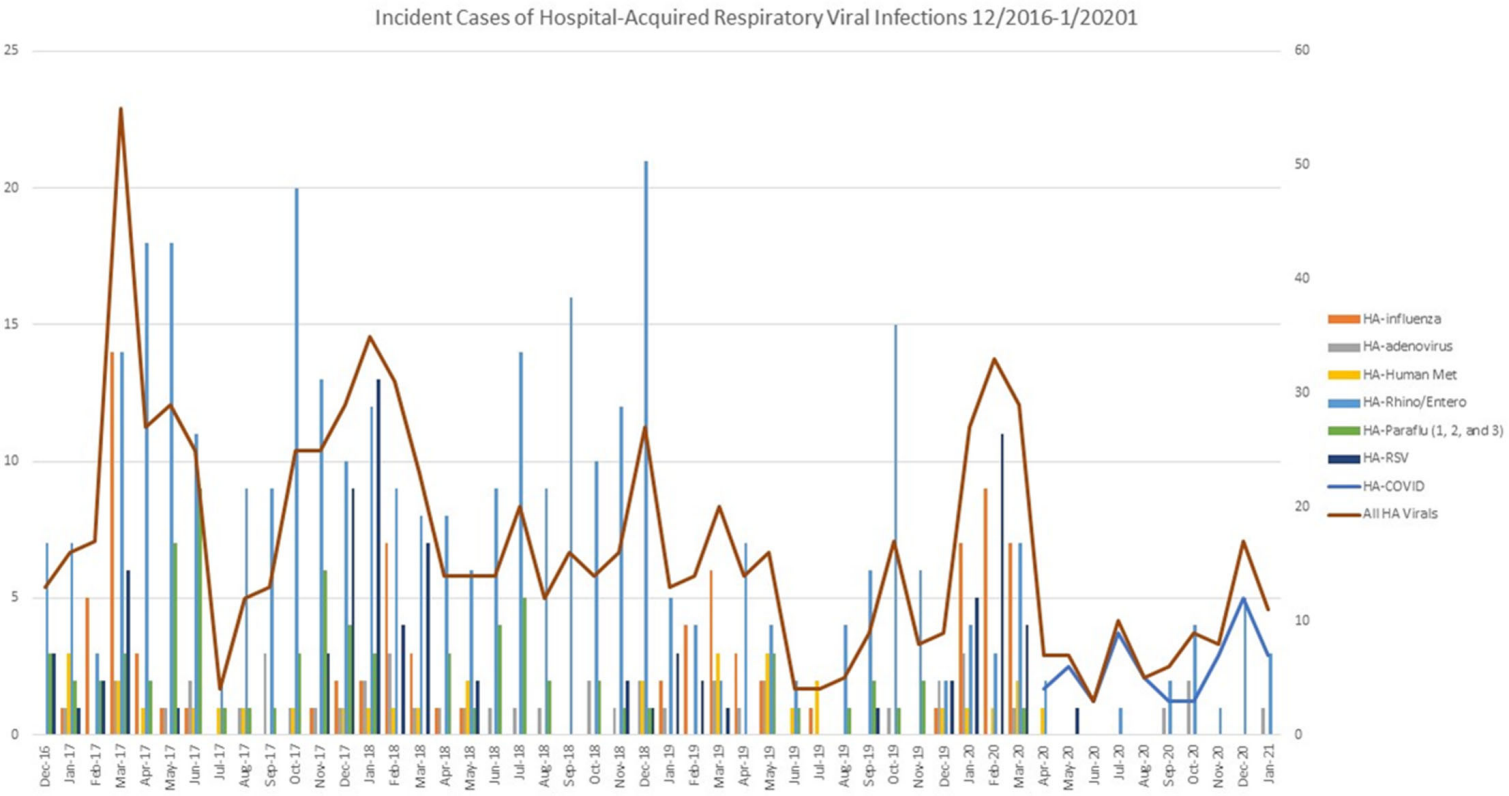# Impact of a Comprehensive SARS-CoV-2 Infection Prevention Bundle on Rates of Hospital-Acquired Respiratory Viral Infections

**DOI:** 10.1017/ash.2021.159

**Published:** 2021-07-29

**Authors:** Jessica Seidelman, Becky Smith, Ibukunoluwa Akinboyo, Sarah Lewis

## Abstract

**Background:** We evaluated the impact of a comprehensive SARS-CoV-2 (COVID-19) infection prevention (IP) bundle on rates of non–COVID-19 healthcare-acquired respiratory viral infection (HA-RVI). **Methods:** We performed a retrospective analysis of prospectively collected respiratory viral data using an infection prevention database from April 2017 to January 2021. We defined HA-RVI as identification of a respiratory virus via nasal or nasopharyngeal swabs collected on or after hospital day 7 for COVID-19 and non–COVID-19 RVI. We compared incident rate ratios (IRRs) of HA-RVI for each of the 3 years (April 2017 to March 2020) prior to and 10 months (April 2020 to January 2021) following full implementation of a comprehensive COVID-19 IP bundle at Duke University Health System. The COVID-19 IP bundle consists of the following elements: universal masking; eye protection; employee, patient, and visitor symptom screening; contact tracing; admission and preprocedure testing; visitor restrictions; discouraging presenteeism; population density control and/or physical distancing; and ongoing attention to basic horizontal IP strategies including hand hygiene, PPE compliance, and environmental cleaning. **Results:** During the study period, we identified 715 HA-RVIs over 1,899,700 inpatient days, for an overall incidence rate of 0.38 HA-RVI per 1,000 inpatient days. The HA-RVI IRR was significantly higher during each of the 3 years prior to implementing the COVID-19 IP bundle (Table 1). The incidence rate of HA-RVI decreased by 60% after bundle implementation. COVID-19 became the dominant HA-RVI, and no cases of HA-influenza occurred in the postimplementation period (Figure 1). **Conclusions:** Implementation of a comprehensive COVID-19 IP bundle likely contributed to a reduction in HA-RVI for hospitalized patients in our healthcare system. Augmenting traditional IP interventions in place during the annual respiratory virus season may be a future strategy to reduce rates of HA-RVI for inpatients.

**Table 1.**
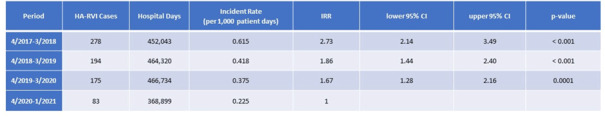

**Figure 1.**